# Time to Exhaustion at the VO_2_max Velocity in Swimming: A Review

**DOI:** 10.2478/v10078-012-0029-1

**Published:** 2012-05-30

**Authors:** Ricardo J. Fernandes, J. Paulo Vilas-Boas

**Affiliations:** 1 CIFI2D, Faculty of Sport, University of Porto, Porto, Portugal.; 2 Porto Biomechanics Laboratory, University of Porto, Porto, Portugal.

**Keywords:** bioenergetics, biomechanics, aerobic power, time limit, training

## Abstract

The aim of this study was to present a review on the time to exhaustion at the minimum swimming velocity corresponding to maximal oxygen consumption (TLim-vVO_2_max). This parameter is critical both for the aerobic power and the lactate tolerance bioenergetical training intensity zones, being fundamental to characterize it, and to point out its main determinants. The few number of studies conducted in this topic observed that swimmers were able to maintain an exercise intensity corresponding to maximal aerobic power during 215 to 260 s (elite swimmers), 230 to 260 s (high level swimmers) and 310 to 325 s (low level swimmers), and no differences between genders were reported. TLim-vVO_2_max main bioenergetic and functional determinants were swimming economy and VO_2_ slow component (direct relationship), and vVO_2_max, velocity at anaerobic threshold and blood lactate production (inverse relationship); when more homogeneous groups of swimmers were analysed, the inverse correlation value between TLim-vVO_2_max and vVO_2_max was not so evident. In general, TLim-vVO_2_max was not related to VO_2_max. TLim-vVO_2_max seems also to be influenced by stroking parameters, with a direct relationship to stroke length and stroke index, and an inverse correlation with stroke rate. Assessing TLim-vVO_2_max, together with the anaerobic threshold and the biomechanical general parameters, will allow a larger spectrum of testing protocols application, helping to build more objective and efficient training programs.

## Introduction

Swimming is a cyclic sport in which both bioenergetical and biomechanical factors assume a fundamental performance-influencing role. Together with running and cycling, swimming has been, along the years, one of the primary areas of research in Sport Sciences, being object of published scientific experimental studies since the 1930s. From the four conventional swimming techniques, front crawl has been the most studied, possibly due to its highest maximal velocity, and to its generalized use in freestyle events and in training. The fact that front crawl is the fastest swimming technique could be explained by its lower intra-cyclic velocity variation, implying lower energy expenditure, and higher propulsive efficiency (di Prampero, 1986; Toussaint and Hollander, 1994; Vilas-Boas et al., 2011).

Once swimming may be considered as an aerobic sport, in which the anaerobic system contribution has significant influence (Capelli et al., 1998; Gastin, 2001; Figueiredo et al., 2011), maximal oxygen consumption (VO_2_max) plays a central role among the energy-yielding mechanisms (di Prampero, 1986); in fact, several authors consider this parameter as the expression of maximal metabolic aerobic performance capability of a subject and, therefore, related to one of the primary areas of interest in swimming training and performance diagnostic (Olbrecht, 2000; Libicz et al., 2005; Rushton, 2007; Sousa et al., 2011). However, and despite the fundamental areas of interest in swimming are already identified (Smith et al., 2002; Rushton, 2007), the study of the maximum duration of exercise in which the intensity corresponding to the minimum velocity that elicits VO_2_max (vVO_2_max) can be maintained is scarcely studied. This parameter, usually denominated as Time Limit (TLim-vVO_2_max), expresses the maintenance of that specific constant velocity to the point of exhaustion, defined by the inability to maintain that precise velocity; so, in the TLim-vVO_2_max assessment, the measure of performance is time duration.

The aim of this paper is to present a review of the literature on this recent topic of interest in swimming. Previously, after the introduction to the topic and the context within which it is proposed, a brief historical overview will be conducted; afterwards, the past research in the area will be examined, highlighting its developments, and the existent studies. Finally, some conclusions will be given, summarizing what has emerged from the literature review, and some suggestions for future studies in this topic will be formulated. To achive these goals, studies were located via computer-generated citations, and a search of key journals and congresses, during October 2011. Two online computer searches, PubMed^™^ and Scopus^™^ databases were conducted to locate published research on Time to exhaustion at the VO_2_max velocity in swimming. The key words used to locate relevant studies in peer reviwed scientific journals, and in the books of the International Symposiums on Biomecanics and Medicine in Swimming were: swimming, swimmer, maximal oxygen uptake, aerobic capacity, aerobic power, time to exhaustion, and time limit. The majority of applicable studies came from the area of exercise physiology (e.g. International Journal of Sports Medicine and European Journal of Applied Physiology).

### Historical approach: TLim-vVO_2_max in treadmill and cycle ergometer

The assessment of TLim-vVO_2_max in swimming was based, and adapted, from earlier studies conducted in treadmill running. To our knowledge, there was a significant temporal gap between the first approach to TLim-vVO_2_max, by Hill and Lupton (1923), in which VO_2_max of running was assessed, and TLim-vVO_2_max estimated (that could be sustained for ∼10 min), and the study of Volkov et al. (1975). These authors used running vVO_2_max to measure the total VO_2_ at that exercise intensity, asking the subjects to maintain that “critical speed” as long as possible (5.4 ± 3.25 min); this parameter should not be confused with the term “critical velocity”, used nowadays for assessing the swimming intensity corresponding to the anaerobic threshold (cf. Smith et al., 2002). Reviewing the literature for this topic, Billat and Koralsztein (1996) found 17 experimental studies published between 1975 and 1995, almost all of them using laboratory procedures, conducting TLim-vVO_2_max tests in special running and cycling ergometers. Afterwards, TLim-vVO_2_max assessment was also applied in rowing and kayaking ergometers (Billat et al. 1996; Hill et al., 2003). From the above-referred studies, two relevant facts were evident: (i) TLim-vVO_2_max appears to give precious information for various matters of training and performance of endurance athletes; and (ii) TLim-vVO_2_max evaluations were accomplished manly in laboratory conditions, using specific ergometers.

### TLim-v V̇O_2_max assessment in swimming

In swimming, the first TLim-vVO_2_max related studies were conducted on a specific ergometer, i.e., in swimming flume (Billat et al., 1996; Faina et al., 1997; Demarie et al., 2001), not in normal swimming-pool conditions; in addition, the study of Demarie et al. (2001) was not performed at vVO_2_max, but at lower exercise intensity: 96% of vVO_2_max. These studies evidenced that TLim-vVO_2_max depended on accumulated oxygen deficit (Faina et al., 1997) and vVO_2_max (Billat et al., 1996; Faina et al., 1997), and that TLim-vVO_2_max was not related with VO_2_max (Billat et al., 1996; Faina et al., 1997). Knowing that, due to the specificity of the physiological demand in swimming, only sports-specific testing provide meaningful results, the evaluation of TLim-vVO_2_max in normal swimming conditions was required.

To our knowledge, Renoux (2001) conducted, for the first time, a TLim-vVO_2_max test in a conventional 25 m swimming-pool, observing that swimmers could sustain that exercise intensity for 6.09 ± 1.39 min; however, this study did not assessed respiratory parameters (e.g. VO_2_ and ventilation), being the vVO_2_max and TLim-vVO_2_max measurements obtained without the confirmation of the major traditional physiological criteria for the achievement of VO_2_max: the occurrence of a plateau in VO_2_ despite an increase in velocity (Howley et al., 1995). In addition, this study evidenced that TLim-vVO_2_max related inversely with vVO_2_max (r = −0.70, p < 0.05).

Once it is accepted, since the final of the 20th century, that the enhancing of swimming performance should no longer be atempted only through the increase of the training volume (Olbrecht, 2000; Rushton, 2007), more objective and specific training sets are required to improve the quality of the swimming training process. Therefore, as the importance of the knowledge on the performance determinant factors, diagnosis methods, and training evaluation and control rised in the last two decades, our group elected TLim-vVO_2_max as an important topic of interest. The relevance of its study was perfectly justified once: (i) TLim-vVO_2_max can be considered as a complementary parameter to VO_2_max and vVO_2_max, the major indicators of maximal aerobic performance, i.e., aerobic power; (ii) TLim-vVO_2_max seems to be a kind of exercise well related to the 400 m front crawl performance, presenting a similar duration and intensity (Termin and Pendergast, 2000); (iii) data on TLim-vVO_2_max, collected in echological swimming conditions, without the possible mechanical constraints of performing in a swimming flume, was needed. So, always testing in swimming-pool conditions, and obtaining physiological and biomechanical data in real time, we tried to answer the following questions: (i) what is the typical duration of the TLim-vVO_2_max effort in front crawl swimming, and would it vary with swimming proficiency and gender? (ii) what is the path of the typical VO_2_ kinetics during a swimming TLim-vVO_2_max exercise, and, as it occurs in the heavy intensity domain, would it be evident a VO_2_ slow component? (iii) TLim-vVO_2_max and VO_2_max are well related in swimming or, as reported for running and cycling, no observable relationship appears? (iv) is TLim-vVO_2_max directly related with two major bioenergetical swimming performance influencing factors - the anaerobic threshold and the energy cost of exercise (C) - and to the swimming general biomechanical influencing factors - stroke rate and stroke length?

**Figure 1:**
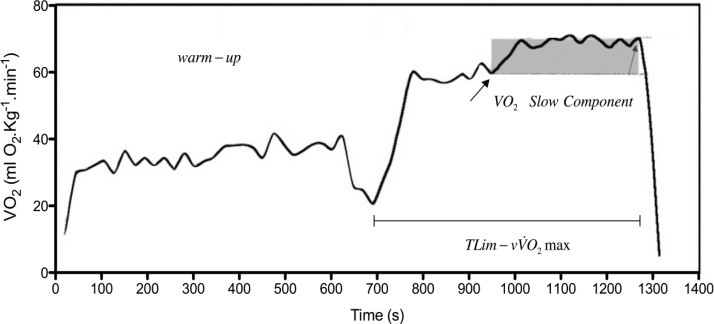
A typical example of the VO_2_pattern during the TLim-vVO_2_max test

To assess TLim-vVO_2_max (and answer to the above-stated questions) it was necessary, in first place, to determine vVO_2_max. Knowing that this exercise intensity is usually assessed trough incremental continuous protocols (Billat and Koralsztein, 1996; Renoux, 2001), Fernandes et al. (2003a) conducted a study for TLim-vVO_2_max characterization, in which an incremental continuous protocol for vVO_2_max assessment was performed. The 10 recreational level swimmers obtained a TLim-vVO_2_max of 5.25 ±1.16 min, situated in between the lower values (4.47 ± 2.40 min) and the higher values (6.15 ± 0.63 min) presented in studies conducted in swimming flume (Billat et al., 1996 and Demarie et al., 2001, respectively). This suggested a lower variation of TLim-vVO_2_max in swimming when compared with the data presented by Billat et al. (1994) for other sports, particularly treadmill running (4–11 min). The inverse relationship between TLim-vVO_2_max and VO_2_max, and vVO_2_max, proposed by Billat et al. (1994) and Billat et al. (1996) for running, and by Billat et al. (1996) and Faina et al. (1997) for swimming, was not observed. In addition, as it is well documented in cycling and running, that exercise at metabolic rates above the anaerobic threshold evidences a slowly-developing component of the VO_2_ kinetics that is superimposed upon the rapid increase of VO_2_ initiated at exercise onset, and that the referred slow increase in VO_2_ continues to rise until the end of the exercise or until exhaustion (Whipp, 1994; Gaesser and Poole, 1996), it was tested the existence of a slow component of VO_2_ kinetics in swimming. In fact, as observed by Demarie et al. (2001) in swimming flume for pentathletes, it was observed a VO_2_ slow component during the TLim-vVO_2_max test in all subjects (279.0 ± 195.2 ml.min^−1^), being its amplitude in agreement with the report of Demarie et al. (2001) (239.0 ± 194.0 ml.min^−1^), but lower than that reported for running and cycling (Billat et al., 1998). In Figure 1, it represented a typical example of the VO_2_kinetics pattern during the TLim-vVO_2_max test, being possible to identify the VO_2_ slow component superimposed after the fast VO_2_ rise. The obtained strong relationship between TLim-vVO_2_max and VO_2_ slow component (r = 0.74, p = 0.01) evidenced that the higher the TLim-vVO_2_max was, the higher the VO_2_ slow component amplitude was expected to be.

However, for the athlete to be able to achieve higher intensity steps in the incremental protocol for vVO_2_max assessment, several researchers from individual sports introduced intermittent progressive protocols for the assessment of that specific exercise intensity. The implementation of (short) rest intervals between steps in the continuous protocol used by Fernandes et al. (2003a), brought some significant improvements in the vVO_2_max assessment methodology: (i) it allowed the swimmer to receive proper feedbacks from the coach and scientific personnel; (ii) swimmers could expel some saliva and condensed that naturally was being accumulated in the mouth piece of the respiratory snorkel and valve system; and (iii) it made possible to collect capillary blood from the ear lobe, allowing assessing, for each swimmer, some fundamental performance determinant parameters, particularly the anaerobic threshold and C. The accurate assessment of the C requires both aerobic and anaerobic energy expenditure evaluation, if possible at different swimming velocities, to allow the computation of an economy curve, which is only possible to be made in a swimming-pool when intervals between steps are implemented.

With this in mind, Cardoso et al. (2003) compared the incremental continuous protocol used by Fernandes et al. (2003a) with the new intermittent incremental protocol for vVO_2_max evaluation, with the same 0.05 m.s^−1^ increments, but including 30 s intervals between steps. No significant differences were observed between protocols in the analysed cardio-respiratory and metabolic parameters, particularly in ventilation (95.3 ± 26.3 vs 95.8 ± 26.6 l.min^−1^), VO_2_max (52.5 ± 9.4 vs 53.4 ± 8.7 ml.kg^−1^.min^−1^), and vVO_2_max (1.16 ± 0.10 vs 1.15 ± 0.10 m.s^−1^), all for p > 0.30. The only difference found was on blood lactate concentration values ([La^−^], 7.36 ± 1.31 vs 8.86 ± 1.93, p = 0.002), but the results were very similar. As well, both protocols fulfilled the requirements of a maximal test for VO_2_max assessment, namely [La^−^] ∼8 mmol.l^−1^, respiratory exchange ratio values >1.0, heart rate >85% of its maximum values, and an exertion to exhaustion (Howley et al., 1995). It was concluded that intermittent incremental protocol was suitable for vVO_2_max assessment in swimming.

Nowadays, the use of the “n × 200 m” intermittent protocol for vVO_2_max assessment is not a new subject in what concerns training control and evaluation of swimmers (Libicz et al., 2005).

Traditionally, VO_2_max assessment protocols in swimming used steps ≥4 min, which are considered most proper for oxygen extraction (Rinehardt et al., 1991). However, following a conventional warm-up, 2–3 min of exercise has been shown to be sufficient time for cardiovascular and biomechanical adaptations to occur, being not observed relevant [La^−^] and VO_2_max differences between incremental protocols of 200, 300 and 400 m step lengths (Fernandes et al., 2011; Fernandes et al., in press). In addition, and not devaluing the necessity to achieve a physiological steady state, the shorter 200 m steps are more specific to the swimming training and competitive requirements, being better accepted by swimmers and coaches.

Fernandes et al. (2003b) conducted another TLim-vVO_2_max related study, aiming to assess it (and VO_2_ slow component) in a higher level sample of swimmers, and using the intermittent protocol for vVO_2_max assessment; to our knowledge, this was the first study that assessed TLim-vVO_2_max and VO_2_ slow component in high level swimmers performing in swimming-pool conditions. Both VO_2_max (76.8 ± 6.5 ml.kg^−1^.min^−1^) and corresponding vVO_2_max (1.46 ± 0.06 m.s^−1^) were higher than the majority of values previously published, perhaps due to differences in the competitive level of the group and/or the testing methodologies used; nevertheless, some studies also reported considerable high values of VO_2_max in high level male front crawl swimmers (Laffite et al., 2004; Sousa et al., 2011). Mean TLim-vVO_2_max value (4.20 ± 1.0 min) was similar to other values reported in flume for competitive swimmers (Billat et al., 1996; Faina et al., 1997), and lower than those obtained with less proficient swimmers (Demarie et al., 2001; Fernandes et al., 2003a). These results, and the inverse relationships between the TLim-vVO_2_max and vVO_2_max (r = −0.47, p < 0.10), and the velocity of anaerobic threshold (r = −0.54, p < 0.05), suggested that the swimmer’s lower level of maximal aerobic metabolic rate might have been associated with a larger capacity to sustain that exercise intensity. This hypothesis was previously pointed out for running (Billat et al., 1994; Billat et al., 1996) and swimming (Billat et al., 1996; Faina et al., 1997; Renoux, 2001), suggesting that the anaerobic capacity can be one of the explanations for this inverse relationship (Billat and Koralsztein, 1996; Faina et al., 1997). However, the correlations of TLim-vV̇O_2_max with [La^−^]max, and Δ[La^−^], were not significant.

In addition, a VO_2_ slow component was also observed (274.1 ± 152.8 ml.min^−1^). Although this value had physiological meaning (once it was higher than 200 ml.min^−1^), it was lower than those presented for running and cycling (Billat et al., 1994), which could be justified by the use of high exercise intensity, the high level of endurance training of these swimmers, and the specificity of this sport. The direct relationship between TLim-vVO_2_max and VO_2_ slow component (r = 0.54, p < 0.05) appeared to indicate that higher TLim-vVO_2_max seems likely to correspond to higher expected VO_2_ slow component amplitude, corroborating previous data in recreational swimmers (Fernandes et al., 2003a), and in other athletes (Whipp, 1994; Gaesser and Poole, 1996). The hypothesis that the VO_2_ slow component phenomenon is related to a major recruitment of fast twitch muscle fibers (with high glycolytic capacity), associated with the fatigue of the previously recruited fibers (Whipp, 1994; Gaesser and Poole, 1996), was not confirmed, corroborating Demarie et al. (2001): no relationship was obtained between VO_2_ slow component and [La^−^]max or Δ[La^−^]in swimming. Nevertheless, it is unlikely that blood lactate *per se* can be responsible for the VO_2_ slow component phenomenon, but rather by accompanying acidosis; this fact allows keeping the suggestion that one of the VO_2_ slow component major contributors is probably related to the superior rates of recruitment of Type II fibers, and additional C of contraction (Whipp, 1994). Another possible contributor for the arising of the VO_2_ slow component may be the increasing ventilation in response to the changes in stroke technique caused by higher levels of fatigue (Demarie et al., 2001). In addition, it is known (cf. Gaesser and Poole, 1996) that, at very high exercise intensities with increased pulmonary ventilation (characteristic of the VO_2_ slow component phase), there is an additional VO_2_ related to the specific work of the respiratory muscles. In fact, Fernandes et al. (2003b) observed a significant correlation between VO_2_ slow component with this additional VO_2_, as well as with the C of the respiratory muscles, suggesting that the ventilatory muscles probably accounts for some, despite low, percentage of the total VO_2_ slow component, as previously mentioned (Whipp, 1994; Gaesser and Poole, 1996).

Knowing that swimming economy is one of the major performance influencing factors (Costill et al., 1985; di Prampero, 1986; Poujade et al., 2002; Smith et al., 2002), Fernandes et al. (2006a) analysed if the net C of swimming affects TLim-vVO_2_max. For that purpose, three swimming economy related parameters were used: the net C corresponding to vVO_2_max (CvVO_2_max), the slope of the regression line obtained from the energy expenditure and corresponding velocities during an incremental test (C_slope_), and the ratio between the energy expenditure mean value and the velocity mean value of the incremental test (C_inc_). Lastly, given that the C differs according to the subjects level (Costill et al., 1985; di Prampero, 1986; Capelli et al., 1998), it was compared the influence of CvVO_2_max, C_slope_ and C_inc_ on the TLim-vVO_2_max in low-level (n=10) and high-level (n=20) swimmers. Both groups presented VO_2_max mean values similar to those previously described: higher values in high-level swimmers (69.9 ± 9.3 ml.kg^−1^.min^−1^), as found in well trained swimmers (Billat et al., 1996; Capelli et al., 1998; Termin and Pendergast, 2000), and moderate values in low-level swimmers (52.1 ± 6.5 ml.kg^−1^.min^−1^), in accordance with the data for recreational and non-specialized swimmers (Costill et al., 1985; Capelli et al., 1998; Demarie et al., 2001; Libicz et al., 2005). As expected, vVO_2_max and the energy expenditure corresponding to vVO_2_max were also higher in the high-level compared with low-level swimmers, reflecting their superior training, proficiency and performance level. TLim-vVO_2_max averaged 3.57 ± 0.91 and 5.13 ± 1.03 min in the high-level and low-level swimmers (respectively), in accordance with the values reported for swimmers of same level (Billat et al., 1996; Demarie et al., 2001; Fernandes et al., 2003a; Fernandes et al., 2003b), and corroborating the distinction between different level athletes in sports in general, and in swimming in particular (Billat and Koralsztein, 1996). TLim-vVO_2_max was inversely related to Cslope, both in total sample and each level groups (r ≥ −0.61, p ≤ 0.05), and to vVO_2_max for the total sample (r = −0.35, p ≤ 0.05), meaning that the swimmers with a worst swimming economy slope profile and vVO_2_max, irrespectively of their performance level, can sustain longer swimming exercises at vVO_2_max; similar results were presented before relating the C and the 400 m swimming distance (Costill et al., 1985; Poujade et al., 2002). As C is obtained by the quotient between energy expenditure and velcocity, and as this fraction is equal to the ratio between drag and propelling efficiency, the above-referred results suggest that technical ability, considered as the result of the latter ratio (di Prampero, 1986; Toussaint and Hollander, 1994; Vilas-Boas et al., 2011), is a fundamental parameter in TLim-vVO_2_max. In fact, the better the swimming technique is, more metabolic power is devoted to move the body forward (overcoming drag), and less is wasted in giving to masses of water a kinetic energy change. However, no relationships between TLim-vVO_2_max and CvVO_2_max, and C_inc_ were observed. The obtained inverse relationship between TLim-vVO_2_max and vVO_2_max is in accordance with the findings of Renoux (2001) and Fernandes et al. (2003b), suggesting that the lower level of maximal aerobic metabolic rate of the less proficient swimmers may be associated with a large capacity to sustain that exercise intensity. It should be realised that the low-level swimmers performed the incremental test at lower absolute velocities than their high-level counterparts, denoting that they could not perform at higher velocities probably due to lower energetic capacity and to lower mechanical efficiency in late test steps (Costill et al., 1985; Toussaint and Hollander, 1994). The reduction in the technical ability due to fatigue in low proficient swimmers is well described (Costill et al., 1985; di Prampero, 1986; Demarie et al., 2001), namely that advanced swimmers are able to swim with a greater distance per stroke than poorer swimmers at a given velocity (Costill et al., 1985; Wakayoshi et al., 1996); this could be due to an enhanced whole body streamlining by the higher level swimmers, which leads to lower frontal surface area and more hydrodynamical global transient body shapes, reducing the hydrodynamic drag forces, and allowing subjects to apply their muscle power to the water effectively through proper technique.

As it is commonly accepted that C, even when related to the body size, depends on gender, and that female swimmers are, in general, more economical than males (Toussaint and Hollander, 1994), Fernandes et al. (2005) analysed if gender has any effect on the relationship between TLim-vVO_2_max and swimming economy. 11 male and 12 female swimmers performed the previously described 7 × 200 m intermittent incremental protocol for vVO_2_max assessment, and CvVO_2_max, C_slope_ and C_inc_ were determined. In addition, as it is known that C is affected by some physical characteristics, it was also studied the influence of body surface area in the C related parameters, and its relationship with TLim-vVO_2_max, by gender. Both male and female groups of swimmers presented VO_2_max mean values (75.1 ± 8.7 and 62.7 ± 5.8 ml.kg^−1^.min^−1^, respectively) similar to those described in the literature for experienced competitive swimmers (Billat et al., 1996; Capelli et al., 1998; Termin and Pendergast, 2000; Fernandes et al., 2003b), and the finding that male swimmers presented higher VO_2_max than female counterparts was also previously described (Costill et al., 1985). As expected, vVO_2_max and energy expenditure at vVO_2_max were higher in the male group, reflecting their higher bioenergetic (VO_2_max) and anthropometric characteristics (body surface area, height and body mass).

In complementarity, the CvVO_2_max mean values were higher for male than female swimmers, which could had some negative effect on their TLim-vVO_2_max results, once it suggests that male specific effort was a more strenuous one that could contribute to an earlier fatigue stage (Billat et al., 1996 and Faina et al., 1997). However, it was not found any difference between genders in the TLim-vVO_2_max exercise (males: 4.04 ± 0.94 min; females: 4.08 ± 1.01 min), as well as any statistical relationship between TLim-vVO_2_max and CvVO_2_max. The fact that [La^−^] were very similar between groups (∼9.5 mmol.l^−1^) could justify, at least in part, the inexistence of differences in TLim-vVO_2_max performances between genders. The TLim-vVO_2_max mean value observed for the male group was similar to the data reported in the literature for male experienced swimmers (Billat et al., 1996; Faina et al., 1997; Renoux, 2001; Fernandes et al., 2003b; Fernandes et al., 2006a); only Demarie et al. (2001) and Fernandes et al. (2003a) presented a higher mean value for this variable, which can be explained by the lower swimming intensity used in their protocol (96% vVO_2_max) and low swimming proficiency level of the subjects, respectively. No previously reports about TLim-vVO_2_max, or at other swimming intensities ∼vVO_2_max, exclusively for female swimmers, were found in the literature. An inverse correlation was found between TLim-vVO_2_max and Cslope for the total sample (r = −0.78, p < 0.001), and for each gender group (r ≥ −0.61, p < 0.05), confirming that swimming economy is a very important performance-influencing factor; these data also evidence that swimmers with a lower swimming economy slope profile, irrespective of their gender, can sustain longer an intensity of swimming corresponding to the vVO_2_max. No significant correlations were found between TLim-vVO_2_max and CvVO_2_max, and C_inc_. As expected, body surface area related positively with C, namely with C_inc_ for the female group of swimmers (r = 0.63, p < 0.05), and in the entire sample of swimmers (r = 0.44, p < 0.05); surprisingly, this relationship did not appear in the male group, suggesting that other parameters than body surface area (e.g. body cross-sectional area, hydrostatic torque, horizontal alignment of the body and body density), can strongly influence C.

To our knowledge, the studies of Fernandes et al. (2006a) and Fernandes et al. (2005) were the first to analyse the relationship between TLim-vVO_2_max and swimming economy. The observed findings confirmed exercise economy as an important factor for swimming performance, evidencing that it should be considered a fundamental parameter of swimming science applied to training (di Prampero, 1986; Toussaint and Hollander, 1994; Smith et al., 2002). In addition, the referred studies have the advantage of having been conducted in swimming-pool, and to focus in the important combination between aerobic and anaerobic metabolic factors of the overall swimming specific metabolic power.

The experimental approaches used in these studies assessed C with the data obtained both from aerobic and anaerobic energy pathways, in opposition to several authors that have determined C by simply estimating the contribution of aerobic metabolism, through the monitoring of VO_2_ at submaximal (or even maximal) intensities (e.g. Costill et al., 1985; Wakayoshi et al, 1996; Poujade et al., 2002).The negligence of the anaerobic contribution to the overall energy requirement in the referred models can be justified by the difficulties imposed by the assessment of the glycolytic system when performing in normal swimming conditions, i.e., in a swimming-pool. However, as TLim-vVO_2_max duration and intensity are closely related to the 400 m front crawl event (Termin and Pendergast, 2000; Fernandes et al., 2003b), in which the anaerobic contribution is ranging between 17 and 40% of the total energy expenditure (Toussaint and Hollander, 1994; Laffite et al., 2004; Gastin, 2001), it was proposed to bridge that difficulty and assess C based on data from aerobic and anaerobic energy pathways. As well, the experiments were conducted in normal swimming-pool conditions, not in swimming flume.

From the results of our group, and from the the literature (Billat et al., 1996; Faina et al., 1997), it is likely that TLim-vVO_2_max performance does not depend directly on the swimmers VO_2_max; in fact, despite the importance of the VO_2_ kinetics in swimming, VO_2_max *de per si* seems not to be considered anymore as one of the main performance determinant factors in this sport (Costill et al., 1985; Toussaint and Hollander, 1994). However, it is not credible to deny that VO_2_max plays a central role among the energy-yielding mechanisms (di Prampero, 1986; Gastin, 2001), and that aerobic capacity is not important for swimming performance; this simply denotes that other factors may obscure the importance of aerobic energy production during swimming, namely in specific TLim-vVO_2_max exercises. As it was observed that the aerobic metabolism did not directly influence the TLim-vVO_2_max, it was hypothesized that the anaerobic performance capacity could be a very relevant parameter to this specific type of exercise, as suggested before (Fernandes et al., 2003b). However, TLim-vVO_2_max did not present any relationship with [La^−^]max and Δ[La^−^] in the TLim-vVO_2_max test; probably, the oxidation of lactate during performance may account for this unexpected result, mainly in expert swimmers, in which the lactate removal ability was found to be higher (Olbrecht, 2000).

For a better knowledge of the complex group of TLim-vVO_2_max determinant factors, Fernandes et al. (2006b) conducted a combined metabolic and biomechanical approach, relating some important technical parameters (stroke rate, stroke length, and stroke index) to TLim-vVO_2_max. The assessment of stroking parameters has been considered relevant for training and performance diagnosis proposes (Keskinen and Komi, 1993; Termin and Pendergast, 2000; Libicz et al., 2005; Figueiredo et al., 2011), being stroke length accepted as a dominant feature of a successful swimming performance (Costill et al., 1985; Craig et al., 1985; Smith et al., 2002; Figueiredo et al., 2011), and stroke index the expression of the swimmer’s ability to move at a given velocity with the fewest number of strokes (Costill et al., 1985). As few studies have related these parameters with the time to exhaustion performed at a specific velocity, 23 highly trained swimmers were studied to observe the existence of a relationship between TLim-vVO_2_max and the stroking parameters. The mean ± SD values of TLim-vVO_2_max (3.53 ± 0.90 min) and vVO_2_max (1.40 ± 0.06 m.s^−1^) were similar to the literature for trained swimmers performing in a flume (Billat et al., 1996; Faina, et al., 1997) and in a normal swimming-pool (Renoux, 2001; Fernandes et al., 2003b). As expected, lower stroke rate and higher stroke length values were obtained in comparison to previous studies that conducted shorter and more intensive swimming events, as using 100 and 200 m distances (Keskinen and Komi, 1993; Figueiredo et al., 2011); similar values were seen in studies that conducted 400 m front crawl tests (Laffite et al., 2004; Alberty et al., 2009). The major findings were the observed inverse relationship between TLim-vVO_2_max and stroke rate (r = − 0.51, p < 0.01), and the direct relationships found between TLim-vVO_2_max and stroke length (r = 0.52, p < 0.01), and stroke index (r = 0.45, p < 0.05), suggesting that swimmers with a higher stroke rate and lower stroke length experienced more difficulties to sustain this maximal aerobic exercise. Indeed, Keskinen and Komi (1993), Wakayoshi et al. (1996) and Alberty et al. (2009) had already noticed a stroke length decrease for exercise intensities higher than the lactate threshold during submaximal constant load tests; a turning point for stroking parameters close to the velocity corresponding to lactate threshold was also observed in an incremental protocol (Fernandes et al., 2011). The capacity to maintain high mechanical propulsive efficiency, i.e., high rates of stroke length and stroke index during the TLim-vVO_2_max, seems to indicate an improved bioenergetic capacity to delay the appearance of increased local muscular fatigue and/or a high capacity to support this situation. In this sense, technical efficiency seems to be a very important influencing factor in TLim-vVO_2_max exercises. TLim-vVO_2_max and vVO_2_max did not present any significant relationship, which is not in accordance with the negative relationships described before (Billat et al., 1996; Faina et al., 1997; Fernandes et al., 2003b; Fernandes et al., 2006a). It is possible that the homogeneity of the sample used by Fernandes et al. (2006b), imposed by skill inclusion criteria, might have diminished the high inter-subject variability described in the above referred studies. As expected, vVO_2_max related positively with stroke length (r = 0.47, p < 0.01), and stroke index (r = 0.72, p < 0.01), expressing that the fastest swimmers were also the more technically proficient. The fact that the fastest swimmers tend to show a smaller decrease in stroke length was previously suggested (e.g. Craig et al., 1985; Smith et al., 2002; Laffite et al., 2004; Figueiredo et al., 2011). So, the more pronounced problems in maintaining stroke length for the less skilled swimmers may be a consequence of a diminished capacity to deliver power output (Toussaint and Hollander, 1994). Perhaps this fact occurs due to a deterioration of body horizontal alignment, which increases drag, and a decrease in the amplitude of the body roll, which consequently induces a decrease in stroke length.

It is well documented that swimming race performance is, among other factors, affected by the strategies swimmers use to control the velocity and the stroking parameters during the various phases of the race (Alberty et al., 2009; Figueiredo et al., 2011). In this sence, Marinho et al. (2006) tried to observe if there were any changes in the stroke rate, stroke length, and stroke index during the course of a typical TLim-vVO_2_max front crawl test (n = 11). As the distances obtained in the TLim-vVO_2_max test were different between swimmers, it were divided in 8 sections to make inter-subjects comparison. It was observed that, in tendency, stroke rate increased and stroke length, and stroke index, decreased during the TLim-vVO_2_max test. When the differences in stroke rate, stroke length and stroke index between each 12.5% section of the test were tested, a significant increase in stroke rate and a decrease in stroke length and stroke index were observed at 25% (74.0 ± 25.8 m), 50% (148.1 ± 51.7 m) and 87.5% (259.2 ± 90.4 m) of the TLim-vVO_2_max test. It was also observed a reduction of stroke length and an increase in stroke rate during the 400 m freestyle event (Keskinen and Komi, 1993; Laffite et al., 2004; Alberty et al., 2009). These results suggest that the changes observed in the stroking parameters in the three points mentioned are critical in the TLim-vVO_2_max exercise; high-speed swimming overloads the human neuromuscular system and may deteriorate the stroke performance during the event, which was already shown in previous studies (Craig et al., 1985; Keskinen and Komi, 1993; Wakayoshi et al., 1996; Laffite et al., 2004; Alberty et al., 2009). The reduction in the mechanical propulsive efficiency is possibly due to the increased local muscular fatigue (Craig et al., 1985; Figueiredo et al., 2011), which seems to reduce the swimmers’ ability to maintain the “feel for the water” (Wakayoshi et al., 1996). This reduction in the quality of stroke technique, represented by the decrease in stroke length and stroke index, and consequent increase in stroke rate to maintain the swimming velocity, is associated with a lower capacity of force production to overcome water resistance (Craig et al., 1985). The data presented by Fernandes et al. (2006b) and Marinho et al. (2006) confirmed that the improvement of stroke length and stroke index, as expressions of technical ability and motor skill, should be promoted and controlled in training; the implementation of training sets that actually increase the ability of the swimmers to maintain their technical proficiency should be daily routine in order to achieve higher mechanical propulsive efficiency in high intensity prolonged exercises.

Lastly, accepting that top-level swimmers have their specificities (Olbrecht, 2000; Smith et al., 2002), it was assessed the TLim-vVO_2_max in elite swimmers, performing in swimming-pool, and analysed its main bioenergetical and biomechanical determinants. Respiratory parameters were measured through a validated telemetric portable gas analyzer (Cosmed K4b^2^, Rome, Italy), which allowed a breath-by-breath collection of data. Considering all sample (3 male and 5 female swimmers), TLim-vVO_2_max ranged from 3.15 to 4.53 min, confirming its low inter-individual variability in swimming, particularly when comparing to running (Billat et al., 1994). However, elite male swimmers performed less time at vVO_2_max than the lower inferior interval value reported by Fernandes et al. (2006a), which seems to be explained by their higher vVO2max, and CvVO2max, when comparing to: (i) elite female swimmers participant in this study; (ii) high trained swimmers (Billat et al., 1996; Renoux, 2001; Fernandes et al., 2003b; Fernandes et al., 2006a); (iii) low level swimmers (Fernandes et al., 2003a; Fernandes et al., 2006a), and (iv) pentathletes (Demarie et al., 2001). Closely related to this finding, it was observed an inverse relationship between TLim-vVO_2_max and vVO_2_max (r = −0.63, P < 0.10), in accordance with previous data (Billat et al., 1996; Faina et al., 1997; Renoux, 2001; Fernandes et al., 2003b; Fernandes et al., 2006a), which seems to be explained by two factors: (i) higher swimming velocities implies superior energy expenditure and, consequently, higher C (Toussaint and Hollander, 1994), confirmed by the high correlation between vVO_2_max and CvVO_2_max (r = 0.74, p < 0.05); and (ii) higher swimming velocities indicates more strenuous efforts, with more pronounced recruitment of anaerobic energy pathways, leading to earlier fatigue stages and, consequently, to lower TLim-vVO_2_max. Fernandes et al. (2008) observed that TLim-vVO_2_max correlated inversely with Δ[La^−^] (r = − 0.69, p = 0.05), and with [La^−^]max (r = −0.63, p < 0.10), confirming the last referred idea, and corroborating the literature (Faina et al., 1997; Billat, 1998; Fernandes et al., 2006a).

In addition, TLim-vVO_2_max was also inversely related to the velocity corresponding to the individual anaerobic threshold(r = −0.62, p < 0.10), which was pointed out before, but only for the averaged value of 3.5 mmol.l^−1^ of [La^−^] (Fernandes et al., 2003b). Knowing that [La^−^] corresponding to AnT has been reported to have great variability between swimmers (Olbrecht, 2000; Fernandes et al., 2011), the methodology for vAnT assessment used by Fernandes et al. (2008) (mathematical modelling of the [La^−^]/velocity curve) was considered more appropriated than the commonly used (linear inter or extrapolation of the [La^−^]/velocity curve), once it gives more specific and individualized values for aerobic/anaerobic transition intensities (Fernandes et al., 2011). In addition, this velocity was highly correlated to vVO_2_max, in accordance to previous available results (Billat et al., 1996; Faina et al., 1997; Renoux, 2001; Fernandes et al., 2006a). Other main TLim-vVO_2_max determinant seems to be VO_2_max slow component; Fernandes et al. (2008) assessed it through mathematical modelling, a more precise and accurate method than the method of the rigid time intervals used by Fernandes et al. (2003a) and Fernandes et al. (2003b). This method is able to discriminate the different components of the VO_2_ kinetics, including the basal, cardiodynamic, fast and slow components, allowing characterizing each one of these components, not only in amplitude, but also in respect to the time of the start of each component; the methods of rigid intervals are not so complete, being not able to assess amplitudes and time delays of the different components of VO_2_ kinetics. The mean value obtained by Fernandes et al. (2008) for the VO_2_ slow component (356.3 ± 168.2 ml.min.^−1^) seems to have physiological meaning, once it was higher than 200 ml.min^−1^, and its significant relationship with TLim-vVO_2_max (r = 0.76, p < 0.05) appears to indicate that higher TLim-vVO_2_max probably corresponds to higher expected VO_2_ slow component amplitude, in accordance with Fernandes et al. (2003b) in high level swimmers.

Lastly, it was shown, once again, that TLim-vVO_2_max do not depend directly on swimmers relative VO_2_max, corroborating previous results from our group and from the literature (Billat et al., 1996; Faina et al., 1997). Despite the indubitable fact that the ability to achieve, and maintain, a specific swimming velocity in an event is related to metabolic but also to biomechanical factors (Toussaint and Hollander, 1994; Termin and Pendergast, 2000), it was not found any relationship between TLim-vVO_2_max and the stroking parameters. It was expected TLim-vVO_2_max to be inversely related with stroke rate and directly related with stroke length and stroke index, as observed by Fernandes et al. (2006b). However, no significant correlation values were obtained. The reduced sample of this study could be one explanation for weak statistical values. Nevertheless, stroke index was strongly related to vVO_2_max (r = 0.79, p < 0.05), meaning that faster swimmers were also the most technically proficient (Costill et al., 1985).

## Conclusions

The main conclusions pointed out that swimmers are able to maintain an exercise intensity corresponding to maximal aerobic power during a temporal interval ranging from 215 to 260 s (elite swimmers), 230 to 260 s (high level swimmers) and 310 to 325 s (low level swimmers). It was not observed any difference in TLim-vVO_2_max performance between genders. It was observed the existence of a VO_2_ slow component during the TLim-vVO_2_max test, in all levels of swimming proficiency, and its magnitude was considered to be physiologically significant (Billat, 2000). TLim-vVO_2_max main bioenergetic and functional determinants were swimming economy and VO_2_ slow component (direct relationship) and vVO_2_max, velocity at anaerobic threshold and Δ[La^−^] (inverse relationship). When analysing more homogeneous groups of swimmers, namely when the subjects were matched by level, the inverse correlation value between TLim-vVO_2_max and vVO_2_max was not so evident. TLim-vVO_2_max seems also to be influenced by stroking parameters, presenting a direct relationship with stroke length and stroke index, and an inverse correlation with stroke rate. In general, TLim-vVO_2_max was not related to VO_2_max.

## Suggestions for future research

Despite the fact that the importance of the study of Biophysics in sports is nowadays well accepted, there is yet a lack of research trying to understand the relationships established between the bioenergetical and biomechanical variables in swimming. In this sense, it is our purpose to continue the study of TLim-vVO_2_max in swimming, namely in the following points: (i) relate all the parameters previously studied with one of the most relevant biomechanical swimming parameter: the intra-cyclic variation of the horizontal velocity of the centre of mass; (ii) assess the distribution of the percentage of energy contribution from each energy system on the TLim-vVO_2_max exercise, i.e., finding which is the aerobic and anaerobic participation percentages, and its specificity across different levels of performance; (iii) knowing that lactate production is not a truly good predictor of performance in swimming, we will look up to new indicators of anaerobic energy system participation like the O_2_ deficit, the lactate exchange ability and the Δ respiratory quotient; (iv) apply the TLim-vVO_2_max to training bout sets of intermittent exercise, and assess the different physiological and biomechanical responses. As secondary goals, it is our aim to increase our knowledge about the VO_2_ kinetics during the intermittent incremental protocol for vVO_2_max assessment. In this sense, we purpose to group the 200 m steps before the occurrence of the individual anaerobic threshold, and the steps that occur after that boundary, and to characterize them. In complementarity, we will observe if the sampling interval of VO_2_ data could affect the prevalence of a plateau in VO_2_ at VO_2_max. Lastly, when relating all the abovementioned parameters, we will try to increase the number of subjects of the samples, in order to be able to use “strongest” and more reliable statistical methods, namely the use of prediction regression models. The increase of the samples size will be also useful to consolidate the data obtained for backstroke, butterfly and breaststroke.
